# Supplementary material for: Chemical Profiling, Bioactive Properties, and Anticancer and Antimicrobial Potential of Juglans regia L. Leaves
Source: Molecules. 2023 Feb 20;28(4):1989. doi: 10.3390/molecules28041989 (PMC9966981; doi:10.3390/molecules28041989)
Supplement: Supplementary file 1 [file molecules-28-01989-s001.zip › molecules-2195462-supplementary.pdf]

molecules

Supplementary material

Chemical Profiling, Bioactive Properties, and Anticancer and Antimicrobial Potential of *Juglans regia* L. Leaves

Natalia Żurek [1], Karolina Pycia [1], Agata Pawłowska [1], Leszek Potocki [2] and Ireneusz Tomasz Kapusta [1,*]

[1] Department of Food Technology and Human Nutrition, College of Natural Sciences, University of Rzeszow, 4 Zelwerowicza St., 35-601 Rzeszow, Poland; nzurek@ur.edu.pl (N.Ż.); kpycia@ur.edu.pl (K.P.); agpawlowska@ur.edu.pl (A.P.)
[2] Department of Biotechnology, College of Natural Sciences, University of Rzeszow, 1 Pigonia St., 35-310 Rzeszow, Poland; lpotocki@ur.edu.pl
* Correspondence: ikapusta@ur.edu.pl; Tel.: +48-17-785-5238

Content:

Supplementary Table S1: Correlation between TPC, TFC, TPA in *J. regia* leaves preparation and antioxidant activity.

Supplementary Table S2: Correlation between TPC, TFC, TPA in *J. regia* leaves preparation and antiproliferation activity

Table S1. Correlation between TPC, TFC, TPA in *J. regia* leaves preparation and antioxidant activity determined by $O_2^{\bullet -}$, OH^-, ChA, ABTS, and CUPRAC methods.

	TPC	TFC	TPA
**$O_2^{\bullet -}$**	-0.992	-0.890	-0.941
**OH^-**	-0.842	-0.733	-0.428
**ChA**	-0.354	-0.182	-0.196
**ABTS**	0.978	0.996	0.993
**CUPRAC**	0.999**	0.998*	0.417

(+) positive correlation, (-) negative correlation, *significant differences at $p < 0.05$, ** significant differences at $p < 0.01$.

Table S2. Correlation between TPC, TFC, TPA in *J. regia* leaves preparation and antiproliferation activity against CCD841 CoN, DLD-1, Caco-2, MCF-7, U87MG, U251MG, and SK-Mel-29 cell lines.

	TPC	TFC	TPA
**CCD841 CoN**	-0.182	-0.354	-0.676
**DLD-1**	-0.161	-0.998*	-0.388
**Caco-2**	-0.333	-0.160	-0.218
**MCF-7**	-0.999*	-0.996	-0.871
**U87MG**	-0.660	-0.515	-0.159
**U251MG**	-0.958	-0.994*	-0.964
**SK-Mel-29**	-0.580	-0.715	-0.923

(+) positive correlation, (-) negative correlation, *significant differences at $p < 0.05$, ** significant differences at $p < 0.01$.
